# Evaluation and validation of commercial antibodies for the detection of Shb

**DOI:** 10.1371/journal.pone.0188311

**Published:** 2017-12-01

**Authors:** Güliz Vanli, Alvaro Cuesta-Marban, Christian Widmann

**Affiliations:** Department of Physiology, University of Lausanne, Lausanne, Switzerland; Indian Institute of Science Education and Research, INDIA

## Abstract

Antibodies are among the most important tools for protein detection but, prior to their usage, proper validation of their appropriateness for given applications is required. The utility of an antibody depends on its sensitivity and specificity. We studied these two aspects in a panel of commercial antibodies against Shb, a platform protein involved in receptor tyrosine kinase signalling, but the function of which is still incompletely understood. Several of the antibodies showed shortcomings or were not acceptable for detection of the endogenous protein. The few that could detect Shb were doing so in either western blotting or immunoprecipitation experiments but a given antibody could not work in both applications. This article provides a resource for the available molecular tools that can be used in future research on Shb.

## Introduction

Shb (SH2 domain-containing adapter protein B) is a 55 kDa adapter protein that is expressed in a wide range of human and mice tissues (http://www.ebi.ac.uk/gxa/genes/ENSG00000107338) [1]. Shb consists of a proline rich motif at the N-terminal that is followed by a phosphotyrosine-binding domain (PTB) and a C-terminal SH2 domain [2]. It can form molecular complexes with its binding partners and modulate various physiological functions including apoptosis, differentiation, proliferation and cytoskeletal alterations [3]. The *in vivo* role of Shb was evaluated by investigating global knock-out (KO) animals. In the C57/BL6 background, absence of Shb is lethal whereas it is viable in mixed 129Sv/C57Bl6/FVB and in BALB/c backgrounds [4]. However, even in the viable strains, the absence of Shb results in severe abnormalities affecting the vasculature, haematopoiesis, reproduction and glucose homeostasis. This indicates that Shb plays key roles during normal development and for homeostasis [3]. At the molecular level, it is poorly understood how Shb mediates these important physiological functions. Therefore, development of tools that allow Shb detection in various experimental conditions is of primary importance. In this study therefore, we have evaluated the specificity of seven commercially available and one custom-made anti-Shb antibodies in recognizing endogenously expressed Shb in accordance with the Western Blotting Minimal Reporting Standards (WBMRS) [5]. Our results show that most of these antibodies are unsuited to detect endogenous levels of the Shb protein and that great caution should be used to interpret the data derived from experiments employing these antibodies.

## Materials and methods

### Cell culture

HEK 293T cells were obtained from the American Type Culture Collection (ATCC) (reference CRL-3216). HEK 293T and CE12 cell lines were kept in DMEM (Invitrogen, catalogue no. 61965) supplemented with 10% fetal bovine serum (Gibco, catalogue no. 10499–044). They were grown at 37°C in a 5% CO_2_ atmosphere and kept at a confluency ranging from 10% (i.e. after splitting) to 80% (i.e. before splitting).

### Generation of CE12 cell line

The HEK 293T-derived CE12 cell line has one of its Shb alleles tagged with the V5 sequence (at the carboxy-terminal end of the protein). The V5 tag was introduced by genome editing using a double nicking CRISPR/Cas9 strategy that minimizes off-target insertions [6]. This approach requires the expression of a Cas9 nickase mutant and a pair of single guide RNAs (sgRNA) in order to introduce targeted single stranded breaks (SSBs) in opposite strands of the genomic DNA [6]. An exogenous single stranded DNA–the ultramer–was provided as a V5-encoding DNA donor to be inserted in the Shb alleles during homology-directed repair of the SSBs. The ultramer contains, at its extremities, two arms of perfect homology to the 6^th^ (last) exon of Shb. The central region of the ultramer bears the V5 sequence [7]. The single guide RNAs and the ultramer were designed using the CRISPR Design web tool (http://crispr.mit.edu/) (Table 1).

**Table 1:** Sequences used to knock-in the V5 epitope in the Shb gene.

	Orientation	Sequence
sgRNA1	3’ →5’	*CCC*TGTGAGCGGACCAGACCTGC
sgRNA2	5’ →3’	CTGAGACTTGGAGGTGCCAG*AGG*
Ultramer		CTACCCATCAAAGGGGCTGAGCACTTGTCCCTCCTCTATCCCGTGGCTG TGAGGACCCTGGGAAAACCAATACCAAATCCACTACTAGGCCTAGACAG TACATGAGCGGACCAGACCTGCCCTGCTCTGTGACAGAGCCTGAGACTT GGAGGTGCCAG**AGA**CCCC

Pam sequences (terminal sequences required for Cas9 recognition) are denoted in italics. The underlined sequence encodes for the V5 tag. The ultramer homology arms match positions 2036–2095 and 2096–2158 of human Shb mRNA (NM_003028.2 NCBI entry). The bold sequence corresponds to a mutated AGG PAM sequence. This mutation ensures that the ultramer, once knocked in the Shb locus, cannot be targeted by the single guide RNAs.

To produce a given sgRNA, two DNA oligonucleotides with complementary sequences were synthetized (Microsynth, Balgach, Switzerland), annealed and subcloned into specific Cas9-encoding vectors. For sgRNA1, the subcloning was performed in pSpCas9n(BB)-2A-Puro (PX462) (Addgene plasmid no. 62987) and for sgRNA2 in pSpCas9n(BB)-2A-GFP (PX461) (Addgene plasmid no. 48140). The cells were then transfected with 2 μg of each plasmid and 1 micromole of the ultramer (chemically synthetized by Integrated DNA Technologies, Coralville, IA) using calcium phosphate-based transfection [8]. Forty-eight hours post-transfection, transiently transfected cells were selected in the presence of puromycin (Thermo, Whaltam, MA) (2000 ng/ml for 200,000 cells in 2 ml) for 24 hours. At the end of the antibiotic selection, single cell clones were generated by limiting dilution and the integration of the V5 tag was assessed by massive parallel sequencing using an Illumina MiSeq sequencer. Briefly, a region within the 6^th^ exon of Shb was amplified by PCR using oligonucleotide #1385 TCGTCGGCAGCGTCAGATGTGTATAAGAGACAGACCAAAGAGAAA TACGTTCTG (underlined sequence: region annealing with residues 1952–1972 of Shb mRNA, NM_003028.2) and oligonucleotide #1386 GTCTCGTGGGCTCGGAGATGTGTATAAGAGACAGCATACACACAACACAAACGAC (underlined sequence: region annealing with residues 2212–2192 of Shb mRNA, NM_003028.2). The non-underlined sequences were used for bar-coding.

### Generation of the Shb^KO^ cell line

The Shb knock-out cell line was derived from CE12 cells. Cas9 nickase was used as described above to generate proximal SSBs, but in this case no ultramer was added. In the absence of an exogenous donor DNA, non-homologous DNA repair leads to the stochastic appearance of insertions and deletions around the SSBs. The sgRNAs used were CCGAGGCGAGCGGCCTTCGCAGC and TCCGCCGCCTCGGCGTCCTGCGG (PAM sequences underlined), located in the 1^st^ exon of Shb and corresponding to positions 664–686 and 713–735 of human Shb mRNA, NM_003028.2). Cells expressing both plasmids were selected and clones were isolated as described above. The presence of insertions or deletions in the different alleles of Shb was assessed by massive parallel sequencing. The amplified region was located in the 1^st^ Shb exon and the oligonucleotides used were TCGTCGGCAGCGTCAGATGTGTATAAGAGACAG CTTCAGCTTGGGCAACAG (underlined sequence: region annealing with residues 592–609 of Shb mRNA, NM_003028.2) and GTCTCGTGGGCTCGG AGATGTGTATAAGAGACAG TCGAAGTCTCGCTCCTTCTG (underlined sequence: region annealing with residues 846–827 of Shb mRNA, NM_003028.2).

### RNAi

CE12 cells were plated at a density of 2 x 10^5^ in 6-well dishes. The next day, the cells were transfected with 40 pmoles of an siRNA targeting Shb (GCACAUGAAACUGGCCAAA, Microsynth) or a control siRNA (AllStars Negative Control siRNA, catalogue no. 1027280; Qiagen, Hilden, DE) using 2 μl of Lipofectamine RNAiMAX (Invitrogen, catalogue no. 13778150) diluted in 200 μl of Opti-MEM (Gibco, catalogue no. 31985062). These siRNA sequences were derived using bioinformatics and research computing online tools from the Whitehead institute (http://sirna.wi.mit.edu). The following day, the culture medium was changed and the cells were subjected to an additional round of transfection. Forty-eight hours after the last transfection, cells were lysed in NP-40 lysis buffer.

### Cell transfection

HEK 293T were plated at a density of 2 x 10^5^ in 6-well plates. One day later, they were transfected with 2 μg of either the Stag-Shb-V5.dn3 plasmid (#927) encoding a Shb version tagged at the N-terminus with the Stag epitope and at the C-terminus with the V5 tag or with the corresponding empty vector (pcDNA3, #1) using a calcium phosphate-based transfection method. Twenty-four hours after the transfection, cells were lysed in NP-40 lysis buffer.

### Western blotting

After being washed two times with ice cold PBS (0.68% NaCl, 0.04% KH_2_PO_4_, 0.15% NaH_2_PO_4,_ pH 7.2), cells were lysed in 1 ml of cold NP-40 lysis buffer (Tris-Cl 50 mM pH 8.0, 150 mM NaCl, 1.0% NP-40). Protein quantification was performed by the Bradford technique. 50 μg of protein for each condition was migrated on a 10% polyacrylamide gel and transferred onto a Trans-Blot nitrocellulose membrane (Bio-Rad catalogue no. 10484060) during 1 hour at 100 Volts. Membranes were blocked in 5% non-fat milk for one hour at room temperature. Primary antibodies were diluted according to the manufacturer’s specifications in 5% non-fat milk in TBS (18 mM HCl, 130 mM NaCl, 20 mM Tris pH 7.2)/0.1% Tween 20 (Table 2). Blots were incubated over-night at 4°C with primary antibodies with gentle agitation. Then, blots were washed with TBS/0.1% Tween 20 for 20 minutes each (x4) and incubated with secondary antibodies for 1 hour at room temperature. Blots were visualized with the Odyssey infrared imaging system (LICOR Biosciences, Bad Homburg, Germany).

**Table 2:** Antibodies tested in this study.

	Name and Catalogue no.	Immunogen	Antibody type	Lot number	Supplier	Concentration/ Dilution
1	Anti-Shb/Shf (C-20): sc-530	C-terminus (last 50 amino acids) of human Shb	Rabbit Polyclonal	F2013	Santa Cruz Biotechnology	2 μg/ml
2	Anti-Shb/Shf (B-1): sc-74483	C-terminal amino acids (380-509) of human Shb	Mouse monoclonal	K0207	Santa Cruz Biotechnology	2 μg/ml
3	Anti-Shb: SAB1300181	Center amino acids (240-275) of human Shb	Rabbit polyclonal	020M0467	Sigma-Aldrich	1 μg/ml
4	Anti-Shb: SAB2104743	N-terminal amino acids (1-51) of human Shb	Rabbit polyclonal	QC27580	Sigma-Aldrich	1 μg/ml
5	Anti-Shb: ab94851	N-terminal amino acids (36-85) of human Shb	Rabbit polyclonal	GR65245-1	Abcam	1 μg/ml
6	Anti-Shb: ab175553	C-terminal amino acids (67-95) of mouse Shb conjugated to keyhole limpet haemocynin (KLH)	Rabbit polyclonal	GR259990-1	Abcam	1/1000
7	Anti-Shb (EPR7976): ab129190	C-terminus of human Shb (amino acids 481-494)	Rabbit monoclonal	GR93075-1	Abcam	1/1000
8	Anti-Shb: ab98007	N-terminal amino acids (1-50) of human Shb	Rabbit polyclonal	GR54367-3	Abcam	1 μg/ml
9	Anti-Shb	The whole recombinant human Shb protein	Rabbit polyclonal	Custom made	Genscript	1 μg/ml
10	Anti-V5: A190-120A	Amino acids (95-108) of RNA polymerase alpha subunit of simian virus 5 conjugated to KLH	Rabbit polyclonal	7	Bethyl	1 μg/ml
11	Anti-V5: 46–1157		Mouse monoclonal	1231236	Thermo-Fisher scientific	1.18 μg/ml
12	Anti-α-tubulin: MCA77G		Rat monoclonal		BioRad Serotec	1/5000
13	Anti-PARP: 9542		Rabbit polyclonal	14	Cell Signaling	1/1000
14	Anti-β-actin: 4970		Rabbit monoclonal	14	Cell Signaling	1/5000
15	Anti-Rabbit antibody Alexa Fluor 680: A-21076		Goat polyclonal	1584296	Thermo-Fisher Scientific	200 ng/ml
16	Anti-Mouse antibody DyLight 800: SA5-10176		Goat polyclonal	RF2212068	Thermo-Fisher Scientific	200 ng/mL

The table lists the antibodies used in the present work, their properties, the providers and how they were employed in Western blotting.

### Immunoprecipitation

Cells were washed with PBS and scraped from Petri dishes in ice-cold PBS, centrifuged and gently resuspended in NP-40 lysis buffer. After 15 minutes of incubation at 4°C with rotation, cellular debris was cleared from the whole cell extracts by centrifugation (13,000 g, 5 minutes). Shb was immunoprecipitated from whole cell lysates by overnight incubation at 4°C with the corresponding antibodies, followed by incubation for 1 hour at 4°C with 20 μl of protein-G coupled magnetic Dynabeads (ThermoFisher, Waltham, MA). The immunoprecipitates were then washed 3 times for 15 minutes with fresh ice-cold NP-40 lysis buffer. Additional aliquots of the whole cell lysates plus the immunoprecipitates were then subjected to SDS-PAGE and western blotting as described in the previous section.

### Subcellular fractionation

Cells were treated with siRNA, as described in the previous section. Cells were then washed once with ice-cold PBS. 440 μL of buffer A (HEPES 10 mM pH 7.9, KCl 10 mM, EDTA 0.1 mM, EGTA 0.1 mM, DTT 1 mM) were added to the cells, which were scraped and transferred to a 1.5 mL tube and kept in ice for 15 minutes. Afterwards, 60 μL of buffer A (containing 5% NP-40 solution) was added to each tube, which was further incubated for 5 minutes on ice. These tubes were briefly vortexed, then nuclei were pelleted by centrifugation (800 g, 5 minutes) and the supernatant (cytoplasmic fraction) was transferred to a new tube. The pelleted nuclei were further washed several times in buffer A to remove contaminating cytoplasmic proteins, while the cytoplasmic fraction was centrifuged again to pellet contaminating nuclei. The nuclei were then lysed by resuspending the pellet in 50 μL buffer B (HEPES 20 mM pH 7.9, NaCl 400 mM, EDTA 1 mM, EGTA 1 mM, DTT 1 mM) and subjected to 4 cycles of vortexing plus a 5 minute-long incubation on ice. Afterwards, unbroken nuclei were pelleted by centrifugation (13,000 g, 5 minutes) and the supernatant (nuclear fraction) was transferred to a new tube.

## Results and discussion

**Fig 1:**
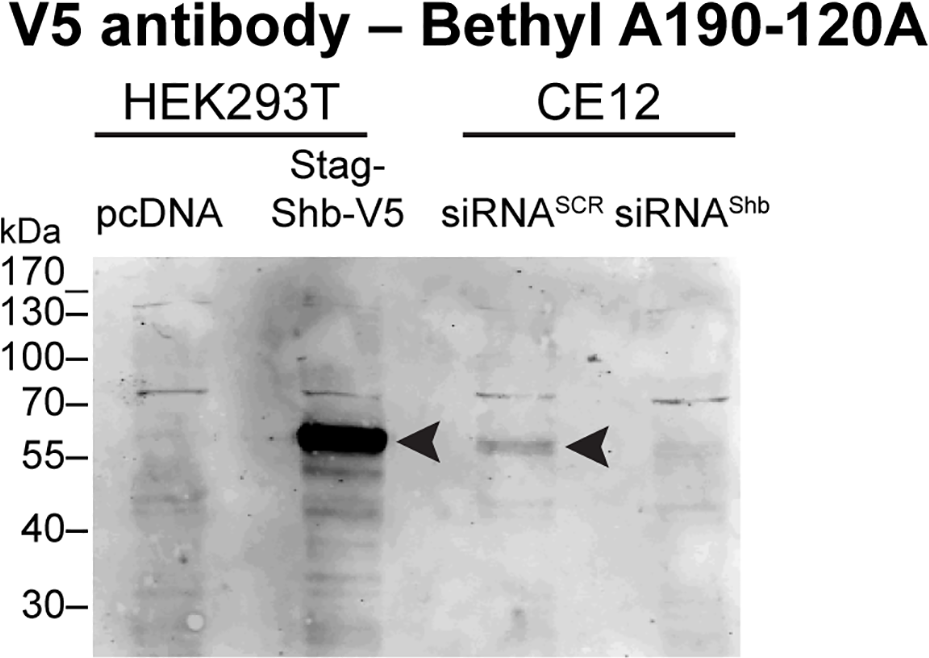
V5-tagged Shb is specifically detected with an anti-V5 antibody. The V5 signal corresponding to Shb-V5 was detected by western blot. HEK 293T cells were transfected with empty plasmid or a Stag-Shb-V5 encoding plasmid. To test the specificity of the signals, the CE12 cell line bearing an endogenous V5-tagged Shb allele was knocked-down for Shb (siRNA^Shb)^ or treated with control siRNA (siRNA^SCR)^. Arrowheads point to Shb-V5. Equal amounts of proteins were loaded (50 μg) on each lane.

We purchased a series of antibodies from various suppliers that were sold as Shb-specific (Table 2). Antibodies targeting conformational epitopes are usually preferred for applications requiring that proteins are kept in their native state such as immunoprecipitation and flow cytometry. In contrast, antibodies targeting linear epitopes are in principle better suited for applications where the proteins are denatured including western blotting, immunocytochemistry and immunohistochemistry. Since the purchased antibodies are falling in these two categories (Table 2), we tested them both by Western blotting and for immunoprecipitation. The HEK 293T cell line was chosen for these analyses because they express Shb at the mRNA and protein levels (ProteomeScout Probeset ID: 204656_at) [9]. Two positive controls were used. The first one corresponds to an over-expression system resulting from HEK 293T cells transiently transfected with a cytomegalovirus (CMV) promoter-driven Stag-Shb-V5 construct. The second one corresponds to an endogenous expression situation where the HEK 293T-derived CE12 cell line has one of its Shb alleles V5-tagged (see the methods). As a negative control, Shb was knocked-down in the CE12 cell line using Shb-specific siRNAs. A shown in Fig 1, a V5-specific antibody efficiently detected over-expressed Shb-V5. Endogenously expressed Shb-V5 was also clearly detected in the CE12 cells. The corresponding band disappeared in the Shb-silenced CE12 cells, demonstrating the specificity of our cellular system.

**Fig 2:**
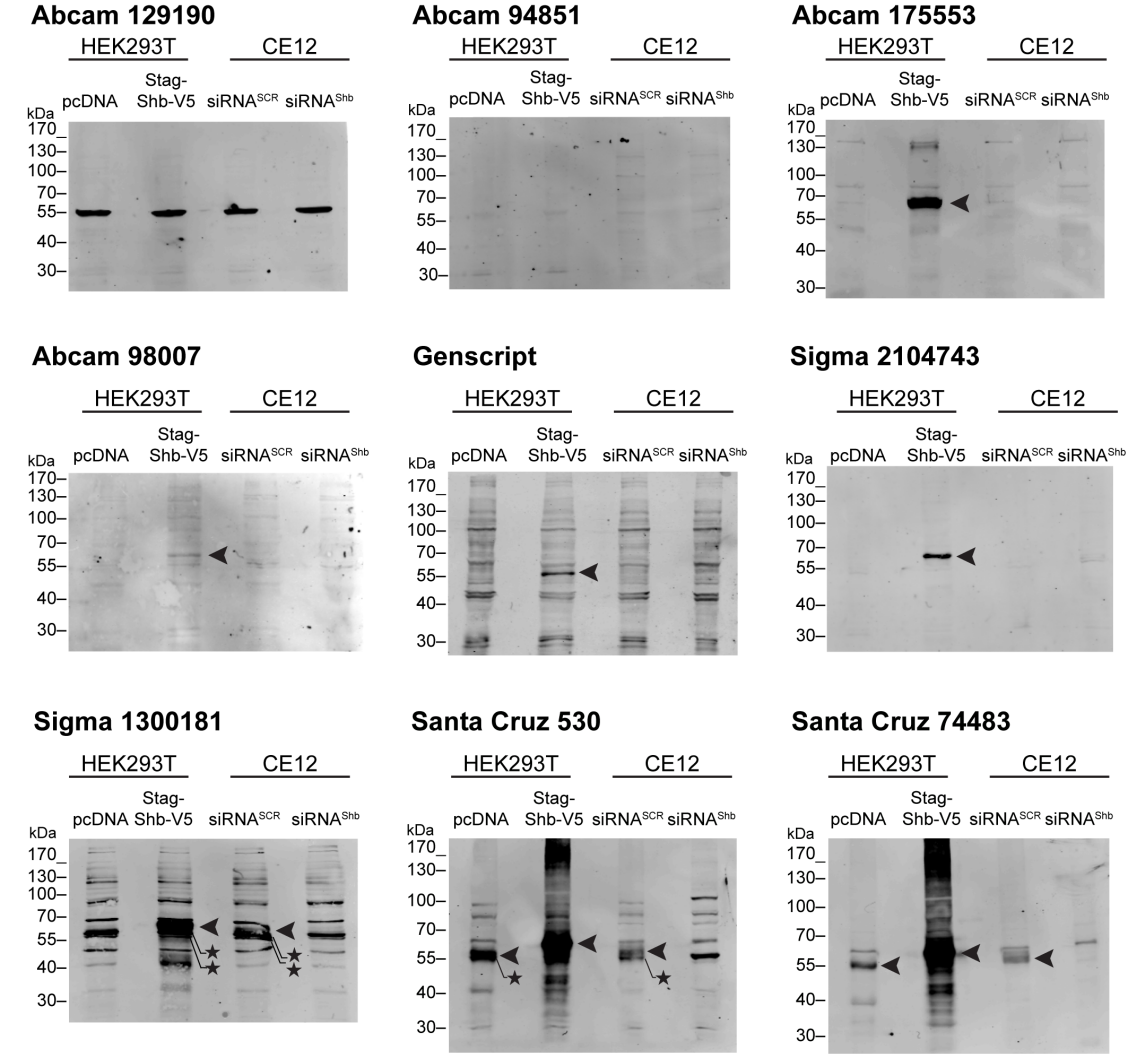
Assessment of nine anti-Shb antibodies. Anti-Shb antibodies were tested by western blotting for their ability to recognize their antigen in HEK 293T cells transfected with either empty plasmid or a Stag-Shb-V5 plasmid, or in CE12 cells, treated with control or Shb-specific siRNA. Whole cell lysates are those that were used in Fig 1. Arrowheads point to Shb-V5; *, unspecific signals.

The Shb antibodies were first tested by Western blotting and the results are reported in Fig 2.

**Abcam 129190.** This antibody recognizes a strong band migrating at around 55 kDa, the expected size of Shb in empty vector-transfected HEK 293T cells. This band, however, neither augmented in Shb-overexpressing cells nor diminished in Shb-silenced cells. Hence, Abcam 129190 is specific for a Shb-unrelated 55 kDa migrating protein.

**Abcam 94851.** This antibody was unable to light up distinct bands on western blots, even though manufacturer’s recommendations were followed. This antibody may have some affinity for the Shb protein but was not shown here. Alternatively, this antibody might not recognize the protein at all.

**Abcam 175553, Abcam 98007, custom-made Genscript, Sigma 2104743.** These four antibodies only detected overexpressed Shb but not endogenous Shb. They are therefore able to recognize Shb but not with much potency (Abcam 98007 was the weakest of all) and consequently cannot be used to study the natural expression of the protein. If only ectopic Shb is to be monitored, Abcam 175553 and Sigma 2104743 can be recommended for their low background detection.

**Sigma 1300181.** This antibody lit up many bands in HEK 293T cells. One of them corresponding to Shb (indicated by an arrow on the blot) because it disappeared in the Shb-silenced cells. This antibody is nevertheless of little use for Shb research because of the extent of non-specific detection, including bands that migrate very close to the protein of interest.

**Santa Cruz 530.** This antibody behaved like Sigma 1300181 but it lit up fewer non-specific bands. Unfortunately, a strong non-specific band was detected very close to Shb, diminishing the usefulness of this antibody for investigating the endogenous protein. It might be potentially a useful antibody to monitor Shb when it is over-expressed though.

**Santa Cruz 74483.** This antibody proved to be the most specific one. It was clearly able to detect the endogenous Shb protein. The strongest signal corresponded to Shb and only one or two non-specific weaker bands were detected.

**Fig 3:**
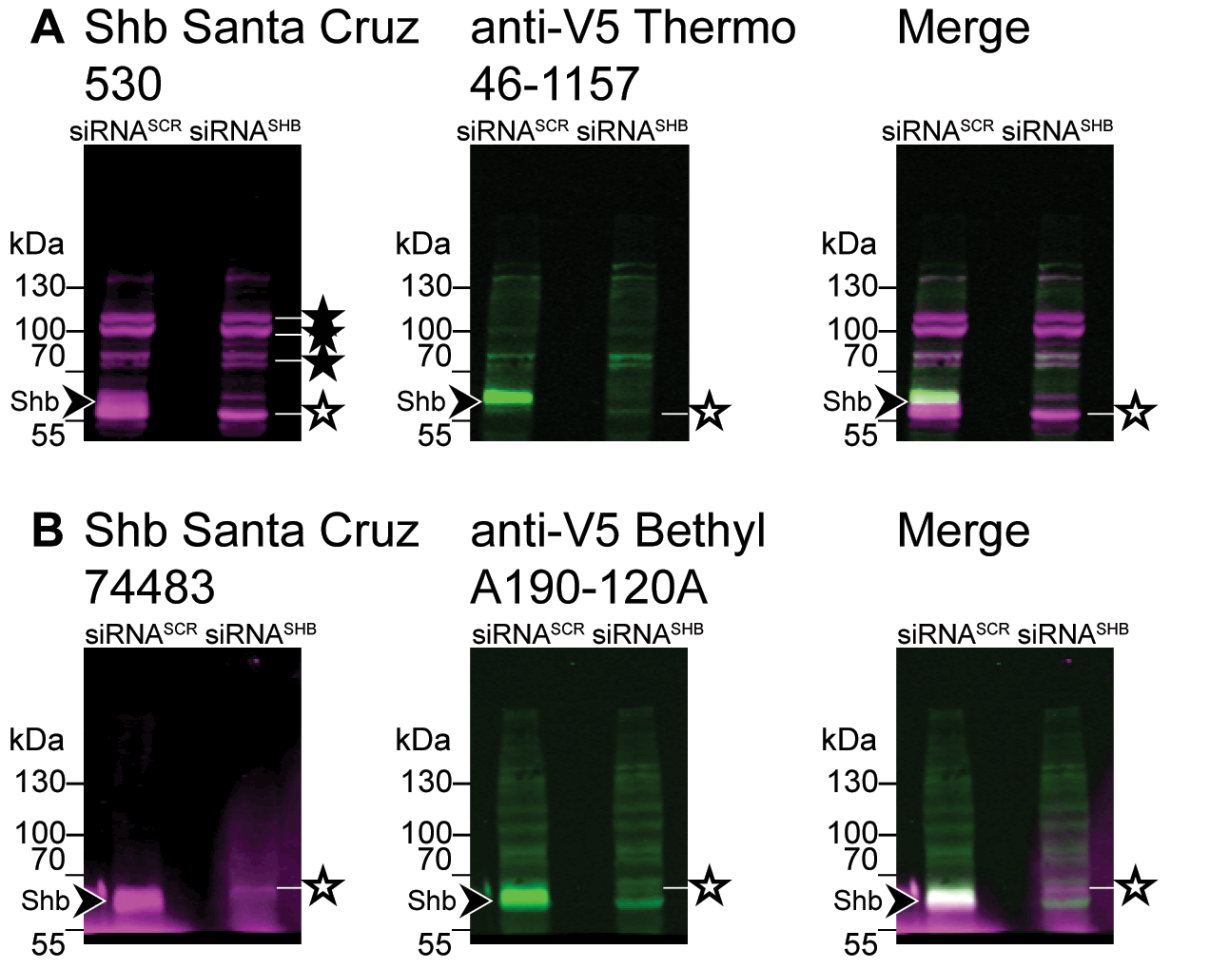
Endogenous Shb-V5 can be detected simultaneously by an anti-V5 antibody and a Shb antibody. CE12 cells, which have one of their Shb allele tagged with V5, were treated with control or Shb-directed siRNAs. Western blots of the cell extracts were performed using simultaneously a Shb-specific antibody (panel A: Santa Cruz 530; panel B: Santa Cruz 74483) and an anti-V5 antibody. The Santa Cruz 530 antibody detected the same band as the anti-V5 antibody (arrowhead) but also many non-specific bands (filled stars), including an intense one in the proximity of the Shb band itself (open star). The Santa Cruz 74483 antibody detected the same band as the anti-V5 antibody (arrowhead) with much more affinity than the closest non-specific band (open star).

To further validate the specificity of these last two antibodies, we performed western blotting using them together with an anti-V5 antibody produced in a different host. The Santa Cruz 530 antibody detected one band which coincided with the band detected by the V5 antibody (Fig 3A), although the non-specific band found closest to Shb obscures its detection. The Santa Cruz 74483 antibody detected a more intense band, which also coincided with the band detected by the V5 antibody (Fig 3B). Although a non-specific band was detected in its proximity, it was much weaker than the Shb band, making it the antibody of choice for western blot application.

**Fig 4:**
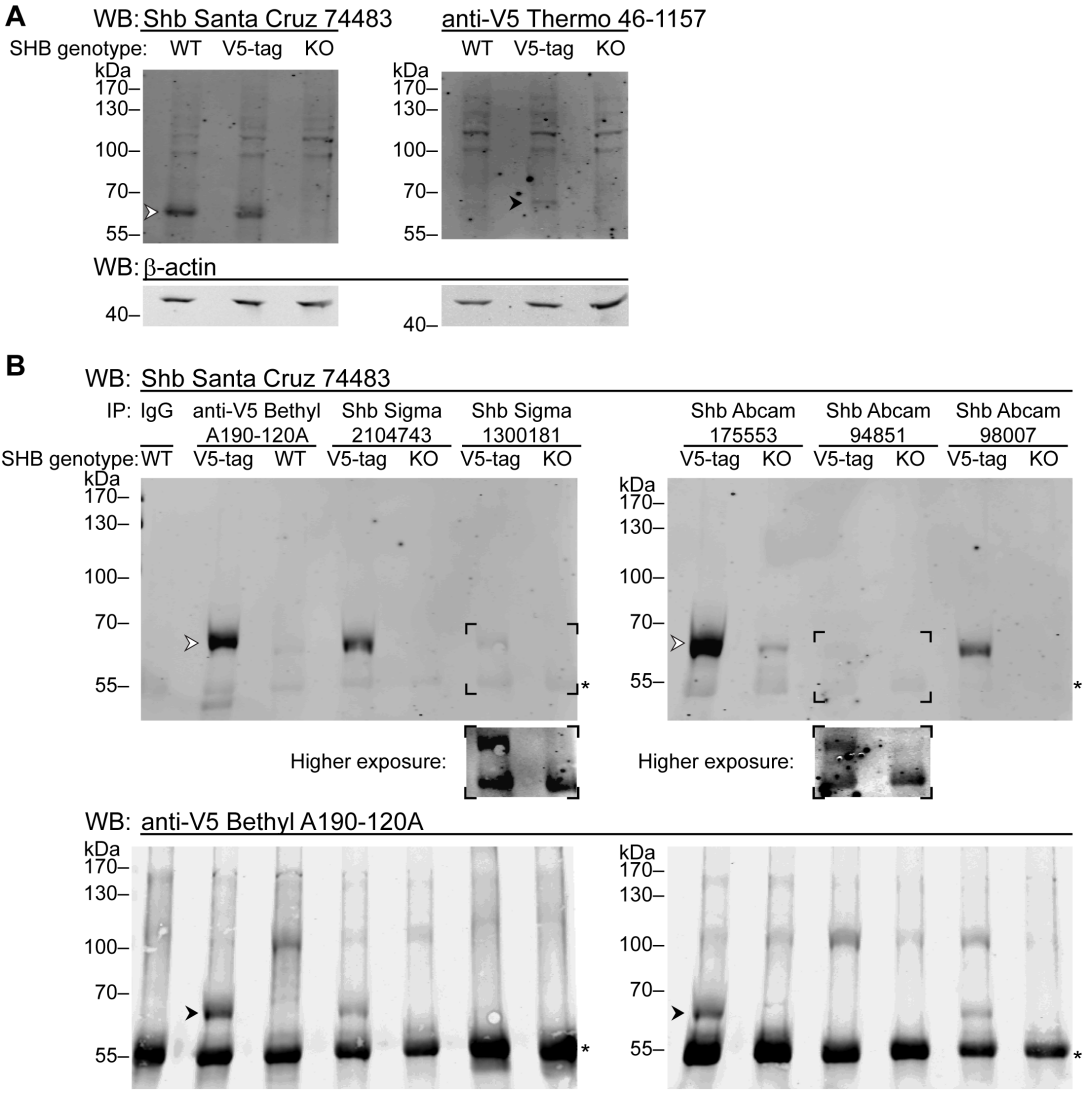
Antibodies that fail to detect Shb in western blot can be used to immunoprecipitate it. Whole cell extracts were prepared from HEK 293T cells encoding endogenous wild-type Shb (WT), HEK 293T CE12 cells having one of their alleles tagged with V5 (V5-tag) or HEK 293T cells knock-out for Shb (KO). **A**: The lysates (40 μg of total protein) were incubated with antibodies against Shb (left panel) or V5-tag (right panel). Arrowheads indicate the band corresponding to Shb. In each case, β-actin was used as a loading control. **B**: Shb was immunoprecipitated from the same whole cell extracts (1.5 mg of total protein) using the six indicated rabbit antibodies. The immunoprecipitates were analysed by western blot using a mouse antibody against Shb (upper panel) and then were reblotted with a rabbit antibody against V5-tag (lower panel). Arrowheads indicate the band corresponding to Shb. The insets show a higher exposure of the areas indicated by the black corners in the upper panel. * indicates the location of the antibody heavy chains, against which the goat secondary antibodies react weakly (in the case of the anti-mouse secondary) or more strongly (in the case of the anti-rabbit secondary). Pre-immune IgG was loaded to allow for the unequivocal identification of the heavy chain.

**Fig 5:**
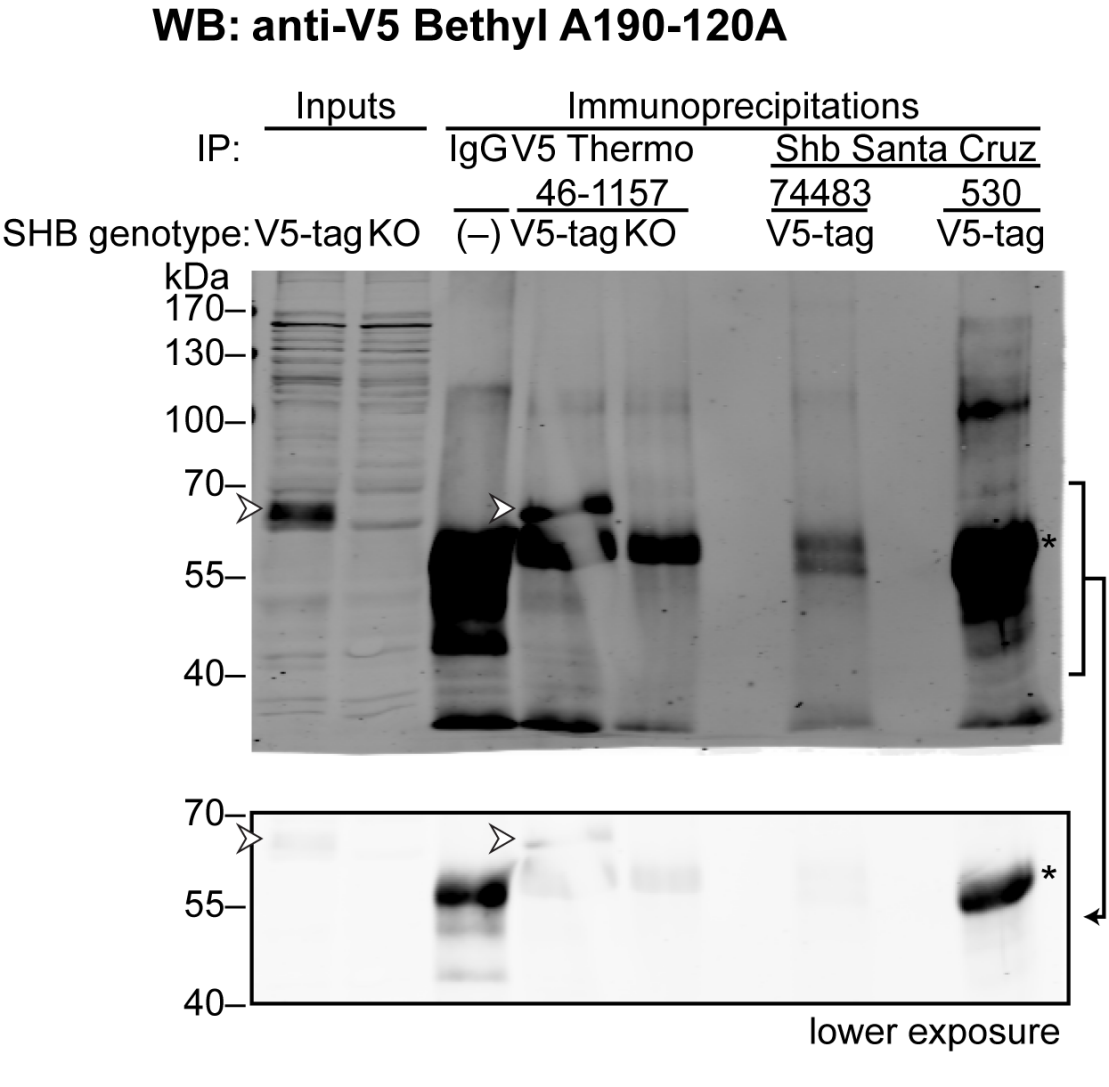
Antibodies that detect SHB in western blot fail to immunoprecipitate it. HEK 293T CE12 cells having one of their alleles tagged with V5 (V5-tag) or HEK 293T cells knock-out for Shb (KO) were used to prepare whole cell extracts, which were then used as material for the immunoprecipitations (40 μg of total protein). Shb was immunoprecipitated from the cell extracts (2 mg of total protein) using the 74483 and 530 antibodies from Santa Cruz. An anti-V5 mouse antibody was used for control immunoprecipitations. The samples were then analysed by western blot using a rabbit anti-V5 antibody. The arrowhead indicates the band corresponding to Shb. The bottom panel shows a lower exposure of the indicated area to better distinguish the heavy chain of the antibody used in the immunoprecipitation (indicated by *), against which the goat anti-rabbit secondary antibodies react (weakly in the case of mouse primary antibodies).

We next tested the performance of the antibodies during immunoprecipitation of cell lysates prepared from HEK 293T, the CE12 clone (which expresses endogenous V5-tagged versions of Shb), and Shb knock-out cells (Fig 4A). The presence of Shb in the immunoprecipitates was assessed by Western blotting using the Santa Cruz 74483 and the anti-V5 antibodies. Fig 4B and Fig 5 report the results of these experiments.

Three antibodies (**Sigma 2104743**, **Abcam 175553** and **Abcam 98007**) were able to immunoprecipitate Shb, identified as a band detected by both the Santa Cruz 74483 and anti-V5 antibodies but absent from the Shb knock-out samples (Fig 4B). The other two antibodies tested, **Sigma 1300181** and **Abcam 94851**, were very poor at immunoprecipitating Shb. Because of their absence of specificity, **Abcam 129190** and **custom-made Genscript** were not tested (see Fig 2).

The ability to recognize endogenous Shb in immunoprecipitation but not during Western blotting suggests that Sigma 2104743, Abcam 175553 and Abcam 98007 may recognize a conformational epitope. Of note, these three antibodies, while not able to reveal endogenous Shb on Western blots, detected over-expressed Shb on membranes. It is possible therefore that a small fraction of over-expressed Shb on Western blot membranes displays the native conformation of the epitopes recognized by these antibodies. Neither 74483 nor 530 from Santa Cruz were able to precipitate Shb in comparable conditions (Fig 5), suggesting that the epitope recognized by these antibodies is indeed only revealed when the protein is denatured.

**Fig 6:**
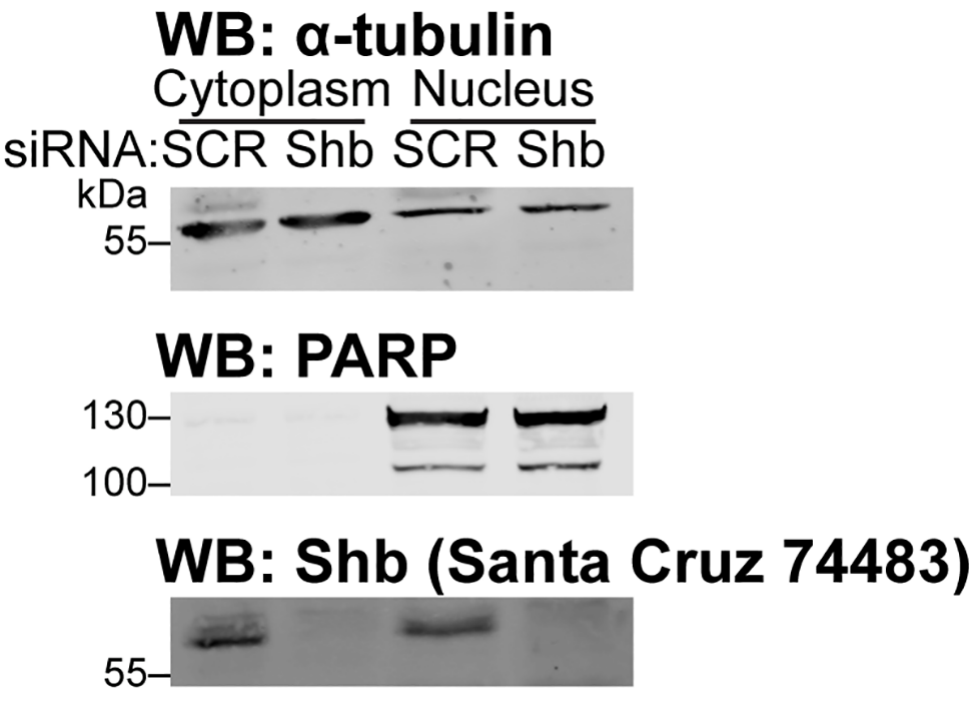
Shb is located in the cytoplasm and in the nucleus. HEK 293T cells, treated with either control or Shb siRNA, were used to isolate nuclear and cytoplasmic cell fractions. These were analysed by western blot using a cytoplasmic marker (α-tubulin), a nuclear marker (PARP) and Shb.

Finally, we decided to use the most specific antibody (Santa Cruz 74483) to determine whether Shb was located in the cytoplasm or in the nucleus. We performed cell fractionation on cells treated with control or Shb-specific siRNAs. Fig 6 shows that the cytoplasmic fraction was devoid of the PARP nuclear marker. The α-tubulin cytoplasmic marker was detected in the nuclear fraction but not as intensely as in the cytoplasmic fraction, suggesting some cytoplasmic contaminants in the nuclear fraction. However, α-tubulin can translocate in the nucleus in some situations [10] and consequently one may underestimate the purity of the nuclear fraction using the α-tubulin marker. In contrast to α-tubulin, Shb was detected with similar intensities in both cytoplasmic and nuclear fractions, suggesting that its presence in the nuclear fraction is not due to a cytoplasmic contamination. Shb has been previously described as a scaffolding protein which regulates tyrosine-kinase signalling through its interactions with PDGFR, FGFR, Fak or Src among others [3]. Its presence in the cytoplasm is therefore expected and confirms an earlier observation [11] regarding its location, which was assessed using immunocytochemistry. The nuclear location of Shb was not anticipated, because canonical nuclear location signals are not found in the Shb sequence. Interestingly, Shb associates with the tyrosine kinase Frk, which has a nuclear location signal and is found in the nucleus [2]. Further studies will be needed to establish how Shb enters the nucleus and the physiological relevance of this translocation.

Antibody validation is an essential step prior to their utilisation, and this becomes especially relevant when the literature describing prior usage is scarce. Here we show that antibodies sold on the market as Shb-specific can be very specific for a protein distinct for Shb (e.g. Abcam 129190), can detect virtually nothing (e.g. Abcam 94851) or can be highly non-specific (e.g. Sigma 1300181). Some were able to recognize Shb but only in certain applications. For example, Santa Cruz 74483 was quite effective at detecting endogenous Shb on Western blot membranes but could not pull-down the protein from cells lysates. Conversely, Sigma 2104743, Abcam 175553 and Abcam 98007 were able to immune-precipitate Shb but could not detect the endogenous protein on membranes.

It is not uncommon for researchers to be confronted with poor results when using commercial antibodies [12–14]. Inadequate antibody validation has led whole research projects to go to waste leading to paper retractions [15] and resources unduly spent. Recent surveys indicate that still too many researchers do not realize the negative impacts that poor antibody validation may have on their research [16]. It is therefore critical to perform thorough validation of antibodies before they are used. The approach employed in this article to test anti-Shb antibodies closely matches the initial part of the Rimm Lab Algorithm for antibody validation [17]. Other approaches can be used such as those relying on proteomic analyses of the bound ligands to validate antibodies for immunoprecipitation purposes for example [18]. Such investigations provide essential resources for specific communities of researchers, who can refer to these results instead of themselves duplicating time- and resource-consuming validation processes. Growing concern about the specificity of commercial antibodies have also prompted the creation of open resources available online [19]. While these not always follow strict criteria for antibody validation, users can still amend or complement the information provided by the retailer of a given antibody for different applications. While this crowdsourced approach to antibody validation may be less rigorous compared to results published in scientific journals, it can provide some retailer-independent antibody validation.

In conclusion, the increasing availability of commercial antibodies makes antibody quality a growing concern. Antibody validation, done by researchers or provided by open access resources, will have to become the norm if results aspire to be trustworthy and reproducible. The data shown here contribute to this effort by reporting information on the quality of commercially available Shb-specific antibodies. This should help researchers planning adequate experiments to study the biological properties of this relatively poorly known protein.
